# Severe and multiple hypoglycemic episodes are associated with increased risk of death in ICU patients

**DOI:** 10.1186/s13054-015-0851-7

**Published:** 2015-04-08

**Authors:** Pierre Kalfon, Yannick Le Manach, Carole Ichai, Nicolas Bréchot, Raphaël Cinotti, Pierre-François Dequin, Béatrice Riu-Poulenc, Philippe Montravers, Djilalli Annane, Hervé Dupont, Michel Sorine, Bruno Riou, on behalf of the CGAO-REA Study Group

**Affiliations:** Service de Réanimation polyvalente, Hôpital Louis Pasteur, CH de Chartres, 34 , avenue du Docteur Maunoury, 28000 Chartres, France; Departments of Anesthesia & Clinical Epidemiology and Biostatistics, Michael G DeGroote School of Medicine, Faculty of Health Sciences, McMaster University, Hamilton, ON Canada; Service de Réanimation médico-chirurgicale, Hôpital Saint-Roch, CHU de Nice, 5, rue Pierre Dévoluy, CS 91179, 06001 Nice Cedex 1, France and IRCAN Unit, UMR INSERM U1081-CNRS 7284, Nice-Sophia Antipolis University, Nice, France; Service de Réanimation médicale, Institut de Cardiologie, CHU Pitié-Salpêtrière, Assistance Publique- Hôpitaux de Paris (AP-HP), 47 bd de l’Hôpital, 75651 Paris cedex 13, France; Service de Réanimation chirurgicale-Brûlés PTMC, Hôtel Dieu, CHU de Nantes, Place Alexis Ricordeau, 44093 Nantes cedex 1, France; Service de Réanimation médicale, Hôpital Bretonneau, CHRU de Tours, 2, boulevard Tonnellé, 37044 Tours cedex 9, France; Service de Réanimation polyvalente, Hôpital Purpan, CHU de Toulouse, Place du Docteur Baylac TSA 40031, 31059 Toulouse cedex 9, France; Département d’Anesthésie et Réanimation chirurgicale, CHU Bichat-Claude Bernard, AP-HP, 46 Rue Henri Huchard, 75018 Paris, France; Service de Réanimation, CHU Raymond Poincaré, AP-HP, 104 Boulevard Raymond Poincaré, 92380 Garches, France; Service d’Anesthésie Réanimation, Hôpital Nord, CHRU Amiens, Place Victor Pauchet, 80054 Amiens Cedex 1, France; Institut National de Recherche en Informatique et en Automatique (INRIA), Domaine de Voluceau, Rocquencourt, B.P. 105, 78153 Le Chesnay, France; Service d’accueil des Urgences, CHU Pitié-Salpêtrière AP-HP, 47 bd de l’Hôpital, 75651 Paris cedex 13, France; Sorbonne Université, UPMC Univ Paris 6, UMR INSERM 1166, Paris, France

## Abstract

**Introduction:**

In a randomized controlled trial comparing tight glucose control with a computerized decision support system and conventional protocols (*post hoc* analysis), we tested the hypothesis that hypoglycemia is associated with a poor outcome, even when controlling for initial severity.

**Methods:**

We looked for moderate (2.2 to 3.3 mmol/L) and severe (<2.2 mmol/L) hypoglycemia, multiple hypoglycemic events (n ≥3) and the other main components of glycemic control (mean blood glucose level and blood glucose coefficient of variation (CV)). The primary endpoint was 90-day mortality. We used both a multivariable analysis taking into account only variables observed at admission and a multivariable matching process (greedy matching algorithm; caliper width of 10^−5^ digit with no replacement).

**Results:**

A total of 2,601 patients were analyzed and divided into three groups: no hypoglycemia (n =1,474), moderate hypoglycemia (n =874, 34%) and severe hypoglycemia (n =253, 10%). Patients with moderate or severe hypoglycemia had a poorer prognosis, as shown by a higher mortality rate (36% and 54%, respectively, vs. 28%) and decreased number of treatment-free days. In the multivariable analysis, severe (odds ratio (OR), 1.50; 95% CI, 1.36 to 1.56; *P* =0.043) and multiple hypoglycemic events (OR, 1.76, 95% CI, 1.31 to 3.37; *P* <0.001) were significantly associated with mortality, whereas blood glucose CV was not. Using multivariable matching, patients with severe (53% vs. 35%; *P* <0.001), moderate (33% vs. 27%; *P* =0.029) and multiple hypoglycemic events (46% vs. 32%, *P* <0.001) had a higher 90-day mortality.

**Conclusion:**

In a large cohort of ICU patients, severe hypoglycemia and multiple hypoglycemic events were associated with increased 90-day mortality.

**Trial registration:**

Clinicaltrials.gov Identifier: NCT01002482. Registered 26 October 2009.

## Introduction

Stress hyperglycemia and insulin resistance are frequent in critically ill patients and associated with poor outcome [1,2]. As compared with permissive hyperglycemia, intensive insulin therapy targeting a tight low range for blood glucose has been shown to reduce in-hospital mortality in a single-center randomized trial in critically ill surgical patients [3]. However, subsequent multicenter randomized controlled trials in broader intensive care unit (ICU) populations either did not find a survival benefit [4-7] or suggested increased mortality [8]. In these studies, a higher prevalence of hypoglycemia was associated with intensive insulin therapy. In patients with diabetes, hypoglycemia is widely recognized as a severe adverse event, leading to neurological insult or death [9,10]. Such association is less clear in the ICU because, in some trials, researchers who have assessed tight blood glucose control observed an increase in hypoglycemia occurrence with [11,12] or without [4-7] a significant increase in mortality. There is a clear association between severity of ICU patients and the occurrence of hypoglycemia [13], and this association may often be underestimated when analyzing the possible link between hypoglycemia and mortality. Moreover, previous studies were associated with methodological concerns, as they included events as covariates that may have been due to hypoglycemia [11,14] or because multivariate analyses may be subject to overfitting due to the multiplicity of cofounders included in the model [15-17]. Therefore, the link between hypoglycemia and mortality, as well as its causality, remains a matter of debate [18].

We used the large cohort of ICU patients included in our recent randomized study [7] to test the hypothesis that hypoglycemia is associated with a poor outcome, even when controlling for initial severity. For this purpose, we used a multivariate analysis taking into account only variables observed at admission. We also used a multivariate matching process, which is less sensitive to unknown bias variables and is considered more powerful, approaching the ideal randomization that is not possible here [19]. We also tested the two other main components of glycemic control (mean blood glucose level and blood glucose variability) that are also suspected to be linked to mortality in ICU patients [20].

## Material and methods

This is an ancillary study of the Computerized Glucose Control in Critically Ill Patients (CGAO-REA) trial (NCT01002482, registered 26 Oct 2009), a non-blinded parallel-group randomized controlled trial involving adult patients admitted to ICUs and comparing tight glucose control with a clinical computerized decision-support systems (blood glucose range between 4.4 and 6.1 mmol/L) and conventional glucose control protocols (blood glucose levels inferior to 10 mmol/L), [7] and corresponds to a post hoc analysis. A subgroup analysis of patients with traumatic brain injury has also been published [21]. The study was approved by an ethical committee (Comité de Protection des Personnes, CCP de Tours, Tours, France). Written informed consent or delayed consent was obtained from each patient or a legal surrogate.

### Patients

Adult patients who were assumed to require at least 3 days in the ICU were eligible for inclusion. We excluded moribund patients with imminent death or those for whom there were do-not-resuscitate orders or the attending physicians were not committed to full supportive care, patients admitted for treatment of diabetic ketoacidosis or hyperosmolar state, patients expected to be eating before the end of the day following the day of admission in the ICU, patients who had previously suffered hypoglycemia without documented full neurological recovery, and patients considered as being at high risk of suffering hypoglycemia. It was not mandatory that the patient experienced an episode of hyperglycemia within 24 hours after admission.

### Study design

Within 24 hours after admission, patients were randomly assigned to undergo tight computerized glucose control or conventional blood glucose control [7]. Blood samples for glucose measurement were obtained by means of arterial catheters whenever possible; the use of capillary samples was discouraged. Blood glucose was measured with bedside glucose readers or preferentially with arterial blood gas analyzer devices when available. At least one blood glucose value per day was measured by the hospital central laboratory on a morning sample (morning laboratory blood glucose). Only the blood glucose values measured at the bedside (for example, either with point-of-care glucose readers or with blood gas analyzers located in the ICU) were used uncorrected for the adaptation of insulin infusion rate. Each ICU used regular human insulin in saline with the same concentration (50 IU in 50 ml of 0.9% sodium chloride) with the use of a pump. A dedicated line for intravenous insulin infusion was encouraged to avoid occult administration of insulin or delay for effective application of a new insulin rate. Enteral feeding was attempted as early as possible. All other aspects of patient care, including nutritional management, were carried out at the discretion of the treating physicians.

At baseline, clinical characteristics, including the Simplified Acute Physiology Score (SAPS II) [22], presence of trauma, the McCabe score for the evaluation of the prognosis of the underlying disease [23] and the Sequential Organ Failure Assessment (SOFA) score [24] were collected.

### Definition of hypoglycemia and blood glucose metrics

Blood glucose control was assessed by morning laboratory blood glucose each day until ICU discharge or day 28. Because different numbers of measures per day were obtained in the two groups, we extracted only four blood glucose measurements per day, closest to 2:00, 8:00, 14:00, and 20:00, respectively. Severe hypoglycemia was defined as blood glucose <2.2 mmol/L, and moderate hypoglycemia was defined as blood glucose between 2.2 and 3.3 mmol/L, as previously described [7]. The number of hypoglycemic events was recorded in each patient, and the density was calculated (expressed as number of events per day of insulin administration and per day in the ICU). We also recorded the mean, minimal and maximal blood glucose readings during the intervention period, the standard deviation (SD) and coefficient of variation (CV) of blood glucose, and the total and daily doses of insulin. Blood glucose variability was considered as moderate (CV, 0.20 to 0.39) or severe (CV, >0.40) as previously described [16].

### Outcome measures

The study primary outcome was death due to any cause within 90 days after ICU admission. Secondary predefined outcomes were death within 28 days after ICU admission, 28-day ICU-free days, 28-day hospital-free days, 28-day ventilator-free days (either mechanical ventilation or non-invasive ventilation), 28-day free-of-catecholamines days, and 28-day free of renal replacement therapy, as previously described [7,25]. These 28-day free-of-treatment days were calculated by subtracting the actual treatment duration in days from 28, with patients who died at day 28 or before being assigned 0 free days. We also compared the following main glucose metrics: mean blood glucose and blood glucose CV [20].

### Statistical analysis

Data are expressed as mean ± SD, median (25th to 75th interquartile range) and number (percentage). Normality was assessed with the D’Agostino-Pearson omnibus test. Comparison of means was performed using Student’s *t*-test or analysis of variance and the Newman-Keuls test. Comparison of medians was performed using the Mann-Whitney *U* test or the Kruskal-Wallis test for multiple comparisons. Comparison of proportions was performed using Fisher’s exact method with the Bonferroni correction when appropriate. Using logistic regression and all variables known at admission to the ICU, we determined variables associated with mortality at 90 days and calculated odds ratios (ORs) and their 95% confidence interval (CIs). Collinearity between variables was considered when *r* >0.60 (Spearman’s matrix correlation coefficient). Calibration of the model was assessed using the Hosmer-Lemeshow test, and discrimination was evaluated using the c-statistic. This model enabled us to calculate the probability of death at 90 days. Then we conducted a multivariate analysis including all significant variables in the model (age, body mass index (BMI), SAPS II score, SOFA score, McCabe score and cause of ICU admission) and by forcing the main variables of blood glucose metrics (severe and moderate hypoglycemia, number of hypoglycemic events (any type, 0 to 2 vs. ≥3), mean blood glucose level, blood glucose CV and type of blood glucose control (conventional vs. computerized)) into the model.

To reduce the effects of confounding, matched populations of patients without hypoglycemia and patients with moderate or severe hypoglycemia were created using propensity score matching. The propensity model was constructed to match pairs of patients without hypoglycemia and hypoglycemic patients using a multivariable logistic model and a greedy matching algorithm with a 1:1 (moderate hypoglycemia) or 2:1 (severe hypoglycemia, multiple hypoglycemic events) ratio and a caliper width of 10^−5^ digit with no replacement. Propensity score matching is one of the propensity score methods used to mimic some of the characteristics of a randomized controlled trial [26]. Applied to the context of our observational study on hypoglycemic events, it consists of forming matched sets of hypoglycemic and non-hypoglycemic patients who share a similar value of the propensity score. In greedy matching within a specified caliper distance, a hypoglycemic patient is first selected at random; the patient without hypoglycemia whose propensity score is closest to that of this randomly selected hypoglycemic patient (with the further restriction that the absolute difference in the propensity scores of matched patients must be below the caliper distance) is chosen for matching to this hypoglycemic patient. This process is called greedy because at each step in the process, the nearest patient without hypoglycemia is selected for matching to the given hypoglycemic patient, even if that patient without hypoglycemia would better serve as a match for a subsequent hypoglycemic patient. This nearest patient without hypoglycemia is then no longer available for a new match with another hypoglycemic patient (no replacement). If no patients without hypoglycemia had propensity scores that lay within the specified caliper distance of the propensity score of the hypoglycemic patient, this unmatched hypoglycemic patient would then be excluded from the matching process. All variables available at admission were included in the matching model. We discarded from the analysis patients without hypoglycemia and hypoglycemic patients for whom no paired match was found. After matching, absolute standardized differences (ASD) were calculated to assess the similarity in preoperative patient characteristics and expressed as a percentage. An ASD >10% was considered to represent a meaningful difference [27]. In order to estimate the association of hypoglycemia and mortality in predefined population subgroups, the same analysis was then conducted in diabetic and non-diabetic patients and in the two groups (conventional vs. computerized blood glucose control).

All *P*-values were two-tailed, and a *P* value <0.05 was considered significant. Statistical analysis was performed using NCSS 7.0 statistical software (NCSS, Kaysville, UT, USA) and R 3.1.1 software (http://www.cran.r-project.org/; The Comprehensive R Archive Network).

## Results

**Figure 1:**
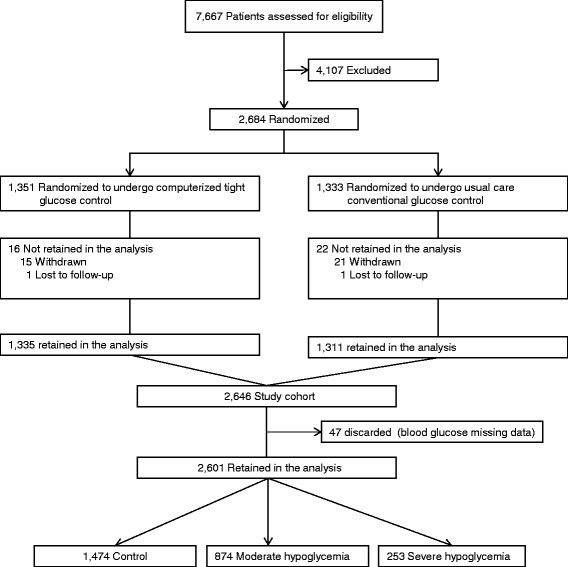
**Flowchart of the study.**

Among the 2,684 randomized patients, 1,351 were assigned to computerized and 1,333 to conventional blood glucose control. Thirty-six patients were discarded from any analysis, thirty-five of whom withdrew consent and one who was included twice. Among the 2,648 remaining patients, 47 were discarded because important data concerning blood glucose measurement were lacking. Thus, 2,601 patients were analyzed (Figure 1).

**Figure 2:**
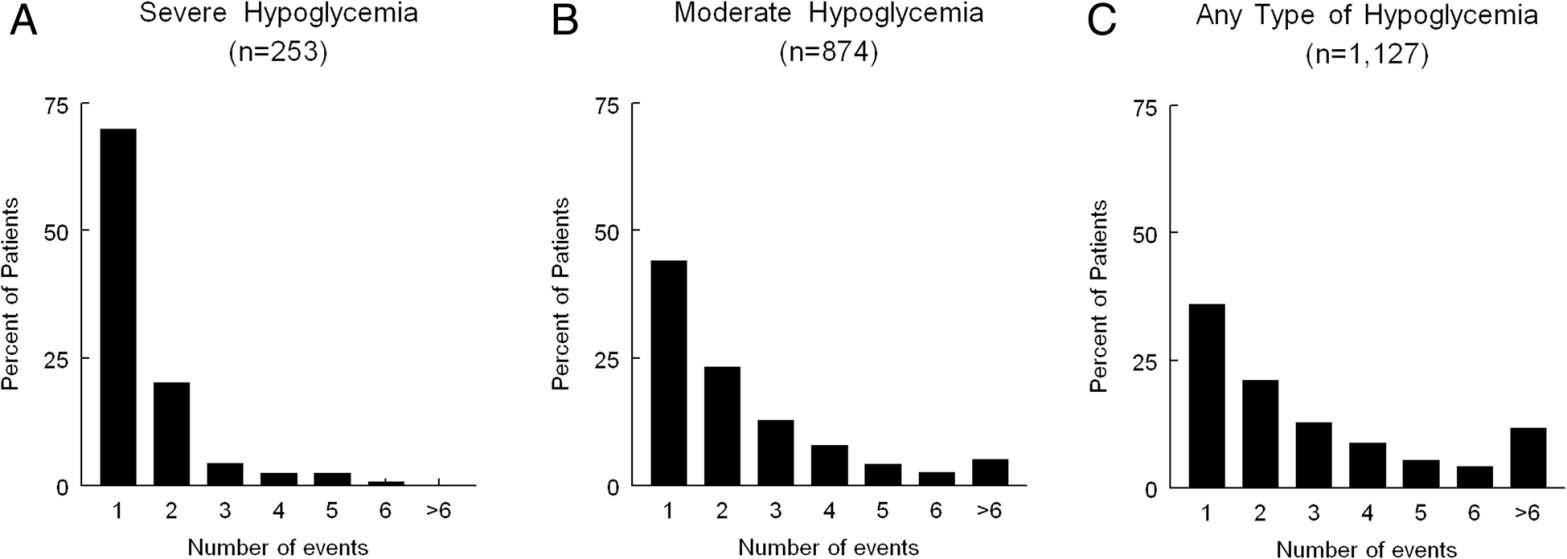
**Distribution of the number of hypoglycemic events per patient. (A)** Severe (<2.2 mmol/L). **(B)** Moderate (2.2 to 3.3 mmol/L). **(C)** Any type (<3.3 mmol/L).

Hypoglycemia (<3.3 mmol/L) occurred in 1,127 patients (43%; 95% CI, 41% to 45%). The density of hypoglycemia was 0.30 events per day of insulin therapy and 0.15 events per day in the ICU. Severe hypoglycemia (<2.2 mmol/L) occurred in 253 patients (10%; 95% CI, 9% to 11%). The density of severe hypoglycemia was 0.037 events per day of insulin therapy and 0.020 events per day in the ICU. Moderate hypoglycemia (2.2 to 3.3 mmol/L) occurred in 874 patients (34%; 95% CI, 32% to 35%). The density of moderate hypoglycemia was 0.26 events per day of insulin therapy and 0.13 events per day in the ICU. Figure 2 shows the distribution of the number of hypoglycemic events per patient (severe, moderate, any type). Multiple hypoglycemic events (any type, n ≥3) occurred in 482 patients (19%; 95% CI, 17% to 20%), and their 90-day mortality was significantly increased compared with patients with fewer hypoglycemic events (51% vs. 29%; *P* <0.001). Blood glucose variability was available in 2,507 patients. Moderate blood glucose variability (CV, 0.20 to 0.39) occurred in 1,447 patients (56%; 95% CI, 54% to 58%), and severe blood glucose variability (CV, ≥0.40) occurred in 238 patients (9%; 95% CI, 8% to 10%). The 90-day mortality was higher in patients with moderate or severe blood glucose variability compared with controls (respectively: 36% vs. 24%, *P* <0.001; 42 vs. 24%, *P* <0.001). There was no significant difference in 90-day mortality between moderate and severe blood glucose variability (*P* =0.21).

Patients were divided into three groups: no hypoglycemia (n =1,474), moderate hypoglycemia (n =874) and severe hypoglycemia (n =253) (Figure 1). The main characteristics of patients in these three groups are shown in Table 1. Patients with moderate or severe hypoglycemia had worse outcomes (Table 1). However, they had more severe illness, as shown by their higher SAPS II and SOFA scores. They had more underlying diabetes and were more frequently mechanically ventilated, on vasopressor therapy or on renal replacement therapy (Table 1). Blood glucose data are shown in Table 2. Patients with moderate or severe hypoglycemia had higher blood glucose variability (SD and CV) and lower mean blood glucose levels (Table 2). Comparable results were noted when considering patients with multiple hypoglycemic events (Table 1).

**Table 1:** **Baseline characteristics and outcome of the study patients** ^**a**^

**Variables**		**No hypoglycemia**	**Moderate hypoglycemia**	**Severe hypoglycemia**	**Multiple hypoglycemic events**
		**(n =1,474)**	**(n =874)**	**(n =253)**	**(n =482)**
Age, yr		61 (17)	63 (16)	62 (15)	63 (15)
Male sex, n (%)		963/1,474 (65)	553/874 (63)	163/253 (64)	320/482 (65)
Weight, kg		80 (21)	75 (18)^b^	74 (20)^b^	74 (18)^b^
Body mass index, kg/m^2^		27.5 (6.7)	26.2 (5.9)^b^	25.6 (6.1)^b^	25.9 (6.0)
SAPS II score		46 (34 to 61)	52 (40 to 67)^b^	59 (46 to 74)^bc^	65 (55 to 75)^b^
SOFA score		7 (4 to 10)	8 (5 to 11)^b^	10 (7 to 13)^bc^	10 (7 to 12)^b^
McCabe score		1 (1 to 2)	1 (1 to 2)	1 (1 to 2)	1 (1 to 2)
Type of patient					
	Medical	872/1,474 (59)	161/874 (60)	161/253 (64)	281/482 (60)
	Scheduled surgery	457/1,474 (31)	75/874 (29)	75/253 (30)	158/482 (30)
	Emergency surgery	145/1,474 (10)	17/874 (11)	17/253 (7)	53/482 (10)
History of diabetes mellitus, n (%)		266/1,474 (18)	189/874 (22)	70/253 (28)^b^	131/482 (27)
	Type 1 diabetes, n (%)	38/1,474 (3)	32/874 (4)	14/253 (6)	22/481 (5)
	Type 2 diabetes, n (%)	228/1,474 (15)	157/874 (18)	55/253 (22)	108/481 (22)
	Not determined				
Previous treatment		118/1,474 (5)	21/873 (8)	21/253 (8)	35/482 (7)
	Insulin, n (%)	114/1,474 (5)	18/873 (7)	18/253 (7)	28/482 (6)
	Antidiabetic drugs, n (%)				
Plasma creatinine, μmol/L		133 (140)	150 (133)	166 (158)^b^	169 (147)^b^
Initial treatment in the ICU					
	Mechanical ventilation	1,273/1,430 (89)	803/873 (92)	240/253 (95)^b^	453/482 (94)^b^
	Catecholamines	708/1,428 (50)	533/253 (61)^b^	195/253 (77)^bc^	353/482 (73)
	Renal replacement therapy	119/1,428 (8)	123/253 (14)^b^	54/253 (21)^bc^	96/482 (20)^b^
	Antibiotics	924/1,428 (65)	597/253 (68)	180/253 (71)	351/482 (73)
Blood glucose control					
	Conventional	900/1,474 (61)	305/874 (35)^b^	79/253 (31)^b^	126/482 (26)^b^
	Computerized	574/1,474 (39)	569/874 (65)^b^	174/253 (69)^b^	356/482 (74)^b^
Treatment-free days					
	Mechanical ventilation	21 (0 to 26)	14 (0 to 23)^b^	0 (0 to 18)^bc^	1 (0 to 18)^b^
	Catecholamines	26 (17 to 28)	23 (0 to 27)^b^	15 (0 to 24)^bc^	18 (0 to 25)^b^
	Renal replacement therapy	28 (21 to 28)	28 (0 to 28)^b^	15 (0 to 28)^bc^	24 (0 to 28)^b^
ICU-free days		20 (0 to 25)	12 (0 to 12)^b^	0 (0 to 17)^bc^	0 (0 to 117)^b^
Mortality					
	Day 28	315/1,474 (21)	222/874 (25)^b^	100/253 (39)^bc^	162/482 (34)^b^
	Day 90	407/1,474 (28)	315/874 (36)^b^	137/253 (54)^bc^	248/482 (51)^b^

^a^Data are expressed as mean (SD), median (25th to 75th interquartile range), and number (%). ICU, Intensive care unit; SAPS II, Simplified Acute Physiology Score II, on which scores can range from 0 to 156, with higher scores indicating more severe illness; SOFA, Sequential Organ Failure Assessment, on which scores can range from 0 to 24 with higher scores indicating more severe illness. ^b^ *P* <0.05 vs. no hypoglycemia group; ^c^ *P* <0.05 vs. moderate hypoglycemia group (comparison was performed only for severe hypoglycemia group).

**Table 2:** **Blood glucose metrics** ^**a**^

**Variables**	**No hypoglycemia**	**Moderate hypoglycemia**	**Severe hypoglycemia**	**Multiple hypoglycemic events**
	**(n =1,474)**	**(n =874)**	**(n =253)**	**(n =482)**
Initial blood glucose, mmol/L	8.9 (4.0)	9.6 (4.6)^b^	9.8 (5.5)^b^	9.7 (4.2)^b^
Mean morning blood glucose, mmol/L	7.2 (2.3)	6.8 (1.6)^b^	6.8 (1.8)^b^	6.7 (1.8)^b^
Mean blood glucose, mmol/L	7.2 (1.7)	6.7 (1.2)^b^	6.8 (1.5)^b^	6.6 (1.2)^b^
Maximum blood glucose, mmol/L	11.3 (1.4)	12.8 (4.1)^b^	14.6 (5.1)^bc^	14.2 (4.9)^b^
Minimum Blood glucose, mmol/L	4.6 (1.4)	2.9 (0.3)	1.7 (0.4)	2.2 (0.6)
Blood glucose standard deviation, mmol/L	1.5 (1.0)	2.0 (1.0)^b^	2.4 (1.3)^bc^	2.2 (1.1)^b^
Blood glucose CV	0.20 (0.15 to 0.26)	0.27 (0.22 to 0.34)^b^	0.32 (0.25 to 0.39)^bc^	0.31 (0.25 to 0.38)^b^
Blood glucose CV >0.20	715/1,428 (50%)	735/873 (84%)^b^	235/253 (93%)^bc^	452/479 (94%)^b^
Total insulin dose, IU	121 (38 to 344)	348 (123 to 801)^b^	442 (129 to 986)^b^	534 (201 to 1109)^b^
Daily insulin dose, IU/day	19 (4 to 41)	34 (15 to 58)^b^	35 (17 to 59)^b^	38 (20 to 61)^b^
Days with insulin	5 (3 to 11)	9 (4 to 17)^b^	10 (5 to 20)^bc^	12 (6 to 22)^b^
Number of blood glucose measurements	28 (16 to 56)	44 (24 to 80)^b^	52 (28 to 104)^bc^	50 (32 to 108)^b^
Number of moderate hypoglycemia		2 (1 to 3)	3 (1 to 6)	4 (3 to 6)
Number of severe hypoglycemia	–	–	1 (1 to 2)	0 (0 to 1)
Number of hypoglycemic events (any type)	–	2 (1 to 3)	5 (3 to 8)	4 (3 to 7)
Hypoglycemic events (any type) ≥3	–	286 (33%)	197 (77%)	482 (100%)

^a^Data are expressed as mean (standard deviation), median (25th to 75th interquartile range) and number (%). CV, Coefficient of variation. ^b^ *P* <0.05 vs. no hypoglycemia group; ^c^ *P* <0.05 vs. moderate hypoglycemia group (only for severe hypoglycemia group). The number of hypoglycemic events and the minimum blood glucose were not tested for statistical significance.

In the multivariable model, six variables were significantly associated with 90-day mortality: age, BMI, SAPS II score, SOFA score, McCabe score and the cause of ICU admission. The discrimination of the model was appropriate (c-statistic =0.745) as well as its calibration (Hosmer-Lemeshow test, χ^2^ =10.12, *P* =0.34). When added to this model, severe hypoglycemia, multiple hypoglycemic events and mean blood glucose level variables were significantly associated with 90-day mortality, in contrast to moderate hypoglycemia and blood glucose CV (Table 3). We next performed a sensitivity analysis by excluding patients allocated to computerized blood glucose control and the variables severe hypoglycemia (OR =2.08; 95% CI, 1.12 to 3.86; *P* =0.02) and mean blood glucose level (OR =1.10; 95% CI, 1.00 to 1.21; *P* =0.045) remained significantly associated with 90-day mortality, whereas the variables moderate hypoglycemia (OR =1.34; 95% CI, 0.94 to 1.91; *P* =0.11) and multiple hypoglycemic events (OR =1.42; 95% CI, 0.85 to 2.37; *P* =0.18) were not.

**Table 3:** **Multivariable analysis of variables associated with 90-day mortality** ^**a**^ **(n =2,312)**

**Variables**		**Odds ratio (95% CI)**	***P*** **-value**
Age (per 1 yr)		1.024 (1.017 to 1.031)	<0.001
Body mass index (per 1 kg/m^2^)		0.979 (0.963 to 0.995)	0.008
SAPS II score (per 1 point)		1.019 (1.012 to 1.025)	<0.001
SOFA score (per 1 point)		1.086 (1.053 to 1.120)	<0.001
McCabe score (per 1 point)		1.587 (1.357 to 1.885)	<0.001
Type of patient			
	Medical	1	–
	Scheduled surgical	0.663 (0.532 to 0.827)	<0.001
	Emergency surgical	0.460 (0.321 to 0.657)	<0.001
Moderate hypoglycemia		1.114 (0.865 to 1.436)	0.40
Severe hypoglycemia		1.571 (1.055 to 2.339)	0.03
Number of hypoglycemic events ≥3		1.834 (1.361 to 2.471)	<0.001
Mean blood glucose (per 1 mmol/L)		1.086 (1.010 to 1.168)	0.03
Blood glucose CV <0.20		1	–
Blood glucose CV 0.20 to 0.39		1.155 (0.906 to 1.472)	0.24
Blood glucose CV ≥0.40		1.041 (0.698 to 1.552)	0.84
Computerized blood glucose control		0.786 (0.639 to 0.958)	0.02

^a^CV, Coefficient of variation; OR, Odds ratio; CI, Confidence interval; c-index =0.757; SAPS, Simplified Acute Physiology Score; SOFA, Sequential Organ Failure Assessment.

Using multivariable matching, we matched each patient with severe hypoglycemia with two patients without hypoglycemia, each patient with moderate hypoglycemia with one patient without hypoglycemia, and each patient with multiple hypoglycemic events with two patients without hypoglycemia. The proportion of discarded patients were 27% in the moderate hypoglycemia group, 21% in the severe hypoglycemia and 51% in the multiple hypoglycemic events groups. After matching, the highest ASD was 5.9%. Table 4 shows the comparison between these matched groups of patients. Although the matching process provided two groups that were comparable for severity, patients with moderate or severe hypoglycemia and those with multiple hypoglycemic events had a poorer prognosis, as shown by a higher 90-day mortality and decreased number of treatment-free days (Table 4).

**Table 4:** **Comparison of outcome and blood glucose metrics in matched groups** ^**a**^

		**Matching for moderate hypoglycemia**			**Matching for severe hypoglycemia**		
**Variables**		**No hypoglycemia**	**Moderate hypoglycemia**	***P*** **-value**	**No hypoglycemia**	**Severe hypoglycemia**	***P*** **-value**
		**(n =640)**	**(n =640)**		**(n =366)**	**(n =199)**	
Probability of death		0.28 (0.19)	0.28 (0.18)	0.66	0.32 (0.19)	0.35 (0.19)	0.12
Outcomes							
	RRT-free days	28 (21 to 28)	28 (7 to 28)	0.06	28 (0 to 28)	24 (0 to 28)	<0.001
	Catecholamine-free days	25 (17 to 27)	24 (2 to 27)	<0.001	24 (0 to 26)	18 (0 to 25)	<0.001
	Mechanical ventilation to free days	20 (0 to 25)	15 (0 to 23)	<0.001	17 (0 to 24)	18 (0 to 25)	<0.001
	ICU-free days	19 (0 to 24)	13 (0 to 22)	<0.001	17 (0 to 24)	0 (0 to 28)	<0.001
	Mortality 28 days	137 (21%)	151 (24%)	0.38	102 (28%)	75 (38%)	0.017
	Mortality 90 days	176 (27%)	213 (33%)	0.029	131 (35%)	106 (53%)	<0.001
Blood glucose metrics							
	Mean blood glucose	7.2 (1.6)	6.6 (1.1)	<0.001	7.3 (1.6)	6.8 (1.5)	<0.001
	Blood glucose CV	0.21 (0.09)	0.28 (0.09)	<0.001	0.22 (0.09)	0.33 (0.12)	<0.001
	Insulin dose (IU/day)	22 (6 to 43)	35 (5 to 59)	<0.001	23 (7 to 41)	35 (19 to 56)	<0.001
		**Matching for multiple hypoglycemic events**					
**Variables**		**No hypoglycemia**	**Multiple hypoglycemic**	***P*** **-value**			
		**(n =445)**	**(n =234)**				
Probability of death		0.32 (0.20)	0.32 (0.19)	0.81			
Outcomes							
	RRT-free days	28 (0 to 28)	28 (0 to 28)	0.06			
	Catecholamine-free days	24 (0 to 26)	19 (0 to 25)	0.001			
	Mechanical ventilation to free days	18 (0 to 24)	5 (0 to 18)	<0.001			
	ICU-free days	17 (0 to 24)	1 (0 to 17)	<0.001			
	Mortality 28 days	115 (26%)	64 (27%)	0.71			
	Mortality 90 days	141 (32%)	108 (46%)	<0.001			
Blood glucose metrics							
	Mean blood glucose	7.0 (1.5)	6.7 (1.0)	<0.001			
	Blood glucose CV	0.24 (0.10)	0.31 (0.09)	<0.001			
	Insuline dose (IU/day)	26 (8 to 49)	41 (22 to 63)	<0.001			

^a^Data are expressed as mean (standard deviation), median (25th to 75th interquartile range) and number (%). ICU, Intensive care unit; RRT, Renal replacement therapy; CV, Coefficient of variation.

**Figure 3:**
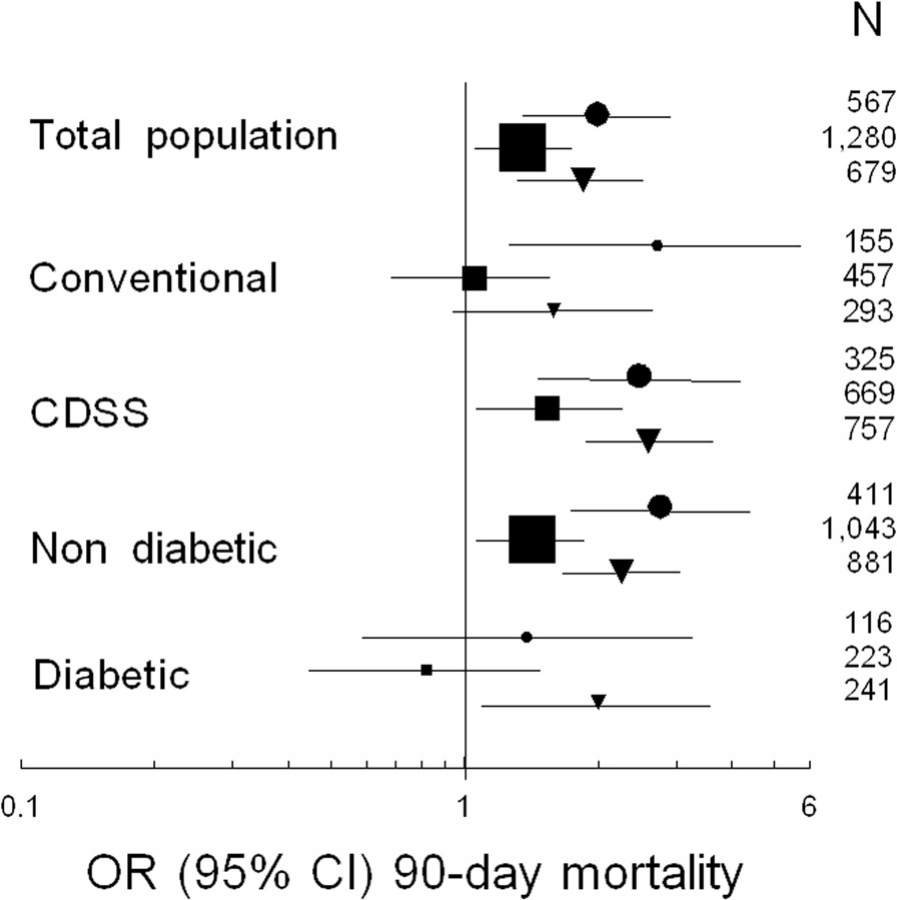
**Subgroup analysis.** Odds ratio (OR) and 95% confidence interval (CI) for death at 90 days associated with moderate (filled squares) or severe (filled circles) hypoglycemia or multiple (n ≥3) hypoglycemic (filled triangles) events in the total population and in matched subgroups according to treatment assignment (conventional vs. computerized decision support system (CDSS)) and diabetes status (diabetic vs. non-diabetic). Matching was performed using propensity score and a ratio of 2:1 for severe hypoglycemia and 1:1 for moderate hypoglycemia and multiple hypoglycemic events. The size of symbols is related to the number of patients (N) retained in the matching process.

The association of severe and moderate hypoglycemia and multiple hypoglycemic events with 90-day mortality was not different among the prespecified subgroup analyses (non-diabetic patients, computerized decision support systems and conventional glucose control), except for patients with diabetes (Figure 3).

## Discussion

In this study, in a large cohort of ICU patients and using two different statistical approaches, we observed that both severe hypoglycemia and multiple hypoglycemic events were associated with increased risk of death. Blood glucose CV was not associated with mortality.

From methodological and clinical points of view, we considered that it was important to perform the best possible adjustment for severity, but using only variables observed at ICU admission. In contrast to some previous researchers [11,28], we did not include any post-ICU admission variable, such as the ICU length of stay, because if hypoglycemia has intrinsic deleterious effects, it should have been able to also impact these variables. We observed that, indeed, hypoglycemia was associated with increased ICU length of stay and use of mechanical ventilation, catecholamines and renal replacement therapy, even when we adjusted for initial severity (Tables 3 and 4). Although many researchers have found an association between hypoglycemia and mortality [11-17,28], others have not [14,29]. Using two different statistical approaches, our study confirms this association between hypoglycemia and mortality, at least when considering patients with severe hypoglycemia and multiple hypoglycemic events (Tables 3 and 4). Matching using propensity score is increasingly reported in the medical literature [30] and has been shown to perform better than multivariable logistic regression in many situations [19]. In our present study, these two approaches lead to consistent estimates of increased 90-day mortality associated with hypoglycemia. The magnitude of the increased risk of death observed in our study is also consistent with previous reports [11,16,17,25,31].

There are three recognized domains of blood glucose metrics—mean blood glucose level, hypoglycemia and blood glucose variability—and all of them have been suspected to be linked to mortality in critically ill patients [13,20]. An important difficulty arises because all these variables are associated with initial severity in ICU patients and are significantly correlated one with the other, mathematically and probably clinically. Moreover, diabetic status is thought to modulate these relationships [28], and the dose of insulin may also be considered as a fourth domain because a high dose of insulin may indicate a high degree of insulin resistance (which is also linked to illness severity) [32] and/or may be associated with non-glucose-related therapeutic effects of insulin [33]. Consequently, there is some debate concerning the respective roles of the different main domains of blood glucose metrics. Our study provides some evidence that blood glucose variability is less important than hypoglycemia. First, we did not observe a dose–response relationship for 90-day mortality with CV, using either a crude or adjusted analysis. Second, after adjusting for initial severity, blood glucose CV was not significantly associated with mortality (Table 3).

Although we performed the best adjustment possible using variables associated with initial severity, a causal relationship between hypoglycemia and mortality cannot be proven in such an observational study [34]. Nevertheless, some arguments are in favor of the existence of a causal relationship in an observational study, at least partially [35]. Among them (strength and consistency of the association, specificity, temporal relationship, plausibility, coherence, experiment and analogy), the Normoglycemia in Intensive Care Evaluation and Survival Using Glucose Algorithm Regulation (NICE-SUGAR) investigators have already evidenced specificity because the association was strongest for death from distributive shock [13]. As previously observed [11,13,14,17,28,31], we observed a dose–response relationship as severe hypoglycemia was associated with higher rates of death than was moderate hypoglycemia or the occurrence of multiple hypoglycemic events. The fact that blood glucose variability did not seem to be associated with mortality is an important point, which reinforces the hypothesis that hypoglycemia (at least severe and/or multiple) is deleterious. It may be also an argument against the hypothesis that the harm of hypoglycemia comes instead from its rapid correction leading to rebound hyperglycemia [13], because this situation is expected to increase blood glucose variability. As previously reported [28], our study also confirms that patients with diabetes may behave differently (Figure 3), but the small sample size of this group precludes definite conclusion.

Our study may enable us to reanalyze our previous published trial on tight blood glucose control, which was characterized by a significant decrease in mean blood glucose with a significant increase in hypoglycemia rate without any impact on mortality [7]. It should be pointed out that such a discrepancy between mortality rate (unchanged) and hypoglycemia rate (increased) was also observed in all previous major trials [4-6], except one [8], excluding the first trial performed during an era without any blood glucose control performed in the control group [3]. This discrepancy was also confirmed by a more recent meta-analysis [36]. Considering that hypoglycemia may be both a marker of severity and a deleterious event, two different hypotheses can be evoked in our trial [7]. First, the benefit of the improvement in blood glucose control may have been masked by the deleterious effects of hypoglycemia. Second, if hypoglycemia is only a marker of illness severity, tight blood glucose may have induced iatrogenic hypoglycemia, which may not be associated with a higher mortality rate. It is not possible to draw definite conclusions, and the two hypotheses may not be exclusive. Anyway, because many arguments suggest that hypoglycemia, at least severe and/or multiple episodes, may be, at least partially, causally associated with mortality, clinicians and caregivers should continue to try to prevent, detect and treat hypoglycemia, particularly when it is severe and/or episodes are repeated. It should be pointed out that we were not able to differentiate iatrogenic (related to insulin administration) and spontaneous hypoglycemic events, whereas the last one is thought to be more strongly associated with mortality, but with possible less of a causal link [37]. Our study provides additional arguments to reduce or suppress hypoglycemic events induced by insulin protocols. Indeed, although most recommendations or guidelines have recently advocated for a higher glucose range target in the ICU based on recent meta-analyses showing a strong association between the glucose range targeted in insulin protocols and the risk for hypoglycemia, our study should lead to optimization of the quality of glycemic control by improving the algorithms embedded in glucose controllers that calculate insulin rates to avoid hypoglycemia, especially in situations where the patient is exposed to variable insulin resistance, rather than by simply increasing the blood glucose target. In other words, minimizing occurrence and recurrence of hypoglycemic events seems more clinically relevant than choosing a high blood glucose target. It has already been demonstrated that an advanced protocol may both decrease the average blood glucose level and reduce the incidence of hypoglycemic events [38]. Further studies are required to develop high-performance glucose controllers in the ICU and to determine the best compromise between individualized blood glucose targets and the risk of hypoglycemia. Finally, whatever the causal link between hypoglycemia and mortality, prevention of hypoglycemic events is needed in the ICU because the occurrence of severe hypoglycemia is *a priori* associated with an increase in nurse workload (*via* additional time required for administering glucose in cases of severe hypoglycemia, which subsequently increases the risk of hyperglycemic rebound and creates a need for more frequent blood glucose monitoring after the hypoglycemic event (in the absence of continuous blood glucose monitoring system)).

In the same manner, our database did not enable us to assess the attributable mortality by incorporating the evolution in severity of illness during the ICU stay and using causal inference methods [39].

The strengths of our study are its size and its prospective and multicentric nature, with a high proportion of patients treated with insulin (98%), a low mean blood glucose level (7.2 mmol/L) within the actual recommended target [40] and a high proportion of patients still in the ICU on the third day (94%). The initial high severity of our patients is shown by the high severity scores (median SAPS II =51, median SOFA =8) and the high mortality rate (33%) [7].

Our study has several limitations. First, sampling of blood glucose was intermittent, and thus it is possible that some patients had undetected hypoglycemia. Second is the absence of data regarding nutritional intake. Third, the fact that our cohort comprises two randomized groups with different methods of blood control may also be considered as a limitation. Nevertheless, the use of a computerized decision support system seemed not to have interfered with our results, except for the role of moderate hypoglycemia because the association between moderate hypoglycemia and mortality was different between the two randomized groups (Figure 3). Fourth, the diabetic status of our patients was not precisely determined using measurement of blood glucose after provoked hyperglycemia or glycated hemoglobin measurement, and thus we did not identify patients with prediabetic or occult diabetic status. In ICU patients, hyperglycemia seems not to be associated with significant increase in mortality in the subgroup of patients with premorbid hyperglycemia [41], but information is lacking with regard to hypoglycemia. Fifth, in ICU patients, severity of hypoglycemic events is usually defined by a biological threshold without a possibility to precisely record clinical signs, and this should also be considered as a limitation, as well as the lack of recording of the duration of the hypoglycemic events and the possible associated rebound in blood glucose levels. Sixth, we did not test all possible metrics of blood glucose variability, but no significant difference has been shown between them [20]. Last, because we included only adult patients, our results may not apply to a pediatric population [42].

## Conclusion

Using two different but concordant statistical approaches in a large cohort of ICU patients, we observed that severe hypoglycemia and multiple hypoglycemic events were associated with increased risk of death at 90 days. In contrast, blood glucose variability was not associated with 90-day mortality.

## Key messages

- Severe hypoglycemia (<2.2 mmom/L) is associated with increased risk of death at 90 days in critically ill patients.
- Multiple hypoglycemic episodes (at least three of any type of hypoglycemia, severe or moderate (2.2 to 3.3 mmol/L)) are associated with increased risk of death at 90 days in critically ill patients.

## Abbreviations

ASD: Absolute standardized difference
BMI: Body mass index
CDSS: Computerized decision support system
CI: Confidence interval
CV: Coefficient of variation
ICU: Intensive care unit
OR: Odds ratio
SAPS II: Simplified Acute Physiology Score II
SD: Standard deviation
SOFA: Sequential Organ Failure Assessment

## References

[1] Dungan KM, Braithwaite SS, Preiser JC (2009). Stress hyperglycaemia. Lancet..

[2] Lena D, Kalfon P, Preiser JC, Ichai C (2011). Glycemic control in the intensive care unit and during the postoperative period. Anesthesiology..

[3] van den Berghe G, Wouters P, Weekers F, Verwaest C, Bruyninckx F, Schetz M (2001). Intensive insulin therapy in critically ill patients. N Engl J Med..

[4] van den Berghe G, Wilmer A, Hermans G, Meersseman W, Wouters P, van Wijngaerden E (2006). Intensive insulin therapy in the medical ICU. N Engl J Med..

[5] Brunkhorst FM, Engel C, Bloos F, Meier-Hellmann A, Ragaller M, Weiler N (2008). Intensive insulin therapy and pentastarch resuscitation in severe sepsis. N Engl J Med..

[6] Preiser JC, Devos P, Ruiz-Santana S, Mélot C, Annane D, Groeneveld J (2009). A prospective randomised multi-centre controlled trial on tight glucose control by intensive insulin therapy in adult intensive care units: the Glucontrol study. Intensive Care Med..

[7] Kalfon P, Giraudeau B, Ichai C, Guerrini A, Brechot N, Cinotti R (2014). Tight computerized versus conventional glucose control in the ICU: a randomized controlled trial. Intensive Care Med..

[8] The NICE-SUGAR Study Investigators (2009). Intensive versus conventional glucose control in critically ill patients. N Engl J Med.

[9] McCoy RG, Shah ND, Van Houten HK, Wermers RA, Ziegenfuss JY, Smith SA (2012). Increased mortality of patients with diabetes reporting severe hypoglycemia. Diabetes Care..

[10] Gerstein HC, Miller ME, Byington RP, Goff DC, Bigger JT, Buse JB (2008). Action to control cardiovascular risk in diabetes study group: effects of intensive glucose lowering in type 2 diabetes. N Engl J Med..

[11] The NICE-SUGAR Study Investigators (2012). Hypoglycemia and risk of death in critically ill patients. N Engl J Med.

[12] Meyfroidt G, Keenan DM, Wang X, Wouters PJ, Veldhuis JD, Van den Berghe G (2010). Dynamic characteristics of blood glucose time series during the course of critical illness: effects of intensive insulin therapy and relative association with mortality. Crit Care Med..

[13] Krinsley JS, Schultz MJ, Spronk PE, Harmsen RE, van Braam Houckgeest F, van der Sluijs JP (2011). Mild hypoglycemia is independently associated with increased mortality in the critically ill. Crit Care..

[14] Arabi YM, Dabbagh OC, Tamim HM, Al-Shimemeri AA, Memish ZA, Haddad SH (2008). Intensive versus conventional insulin therapy: a randomized controlled trial in medical and surgical critically ill patients. Crit Care Med..

[15] Egi M, Bellomo R, Stachowski E, French CJ, Hart GK, Taori G (2010). Hypoglycemia and outcome in critically ill patients. Mayo Clin Proc..

[16] Hermanides J, Vriesendorp TM, Bosman RJ, Zandstra DF, Hoekstra JB, Devries JH (2010). Glucose variability is associated with intensive care unit mortality. Crit Care Med..

[17] Mowery NT, Guillamondegui OD, Gunter OL, Diaz JJ, Collier BR, Dosset LA (2010). Severe hypoglycemia while on intensive insulin therapy is not an independent predictor of death after trauma. J Trauma..

[18] Finfer S (2011). Hypoglycemia in critically ill adults - association yes, causality not proven. Crit Care..

[19] Cepeda MS, Boston R, Farrar JT, Strom BL (2003). Comparison of logistic regression versus propensity score when the number of events is low and there are multiple confounders. Am J Epidemiol..

[20] Mackenzie IMJ, Whitehouse T, Nightingale PG (2013). The metrics of glycaemic control in critical care. Intensive Care Med. 2011;37:435–43. A published erratum appears in. Intensive Care Med..

[21] Cinotti R, Ichai C, Orban JC, Kalfon P, Feuillet F, Roquilly A (2014). Effects of tight computerized glucose control on neurological outcome in severely brain injured patients: a multicenter sub-group analysis of the randomized-controlled open-label CGAO-REA study. Crit Care..

[22] Le Gall JR, Lemeshow S, Saulnier F (1993). A new Simplified Acute Physiology Score (SAPS II) based on a European/North American multicenter study. JAMA..

[23] McCabe WR, Jackson GG (1962). Gram-negative bacteremia: I. Etiology and ecology. Arch Intern Med..

[24] Vincent JL, Moreno R, Takala J, Willatts S, De Mendonça A, Bruining H (1996). The SOFA (Sepsis-related Organ Failure Assessment) score to describe organ dysfunction/failure. Intensive Care Med..

[25] Rubenfeld GD, Angus DC, Pinsky MR, Curtis JR, Connors AF, Bernard GR, the Members of the Outcomes Research Workshop (1999). Outcomes research in critical care: results of the American Thoracic Society Critical Care Assembly Workshop on Outcomes Research. Am J Respir Crit Care Med.

[26] Austin PC (2011). An introduction to propensity score methods for reducing the effects of confounding in observational studies. Multivariate Behav Res..

[27] Austin PC (2011). Optimal caliper widths for propensity-score matching when estimating differences in means and differences in proportion in observational studies. Pharm Stat..

[28] Krinsley JS, Egi M, Kiss A, Devendra AN, Schuetz P, Maureer PM, et al. Diabetes status and the relation of the three domains of glycemic control to mortality in critically ill patients: an international multicenter cohort study. Crit Care. 2013;17:37. 10.1186/cc12547 PMC3733432 23452622

[29] Vriesendorp TM, van Santen S, DeVries JH, de Jonge E, Rosendaal FR, Schultz MJ (2006). Predisposing factors for hypoglycemia in the intensive care unit. Crit Care Med..

[30] Gayat E, Piracchio R, Resche-Rigon M, Mebazaa A, Mary JY, Porcher R (2010). Propensity scores in intensive care and anaesthesiology literature: a systematic review. Intensive Care Med..

[31] Krinsley JS, Grover AG (2007). Severe hypoglycemia in critically ill patients: risk factors and outcomes. Crit Care Med..

[32] Huang CL, Wu YW, Hsieh AR, Hung YH, Chen WJ, Yang WS (2013). Serum adipocyte fatty acid-binding protein levels in patients with critical illness are associated with insulin resistance and predict mortality. Crit Care..

[33] Van den Berghe G (2004). How does blood glucose control with insulin save lives in intensive care?. J Clin Invest..

[34] Goodman SN (1999). Toward evidence-based medical statistics. 1: the *P* value. Ann Intern Med.

[35] Hill AB (1965). The environment and disease: association or causation?. Proc R Soc Med..

[36] Griesdale DE, de Souza RJ, van Dam RM, Heyland DK, Cook DJ, Malhotra A (2009). Intensive insulin therapy and mortality among critically ill patients: a meta-analysis including NICE-SUGAR study data. CMAJ..

[37] Kosiborod M, Inzucchi SE, Goyal A, Krumholz HM, Masoudi FA, Xiao L (2009). Relationship between spontaneous and iatrogenic hypoglycemia and mortality in patients hospitalized with acute myocardial infarction. JAMA..

[38] Chase JG, Shaw G, Le Compte A, Lonergan T, Willacy M, Wong X (2008). Implementation and evaluation of the SPRINT protocol for tight glycaemic control in critically ill patients: a clinical practice change. Crit Care..

[39] Bekaert M, Timsit JF, Vansteelandt S, Depuydt P, Vésin A, Garrouste-Orgeas M (2011). Attributable mortality of ventilator-associated pneumonia: a reappraisal using causal analysis. Am J Respir Crit Care Med..

[40] Ichai C, Preiser JC, for the Société Française d’Anesthésie-Réanimation (SFAR)3 and Société de Réanimation de langue Française (SRLF) and the Experts group (2010). International recommendations for glucose control in adult non diabetic critically ill patients. Crit Care.

[41] Plummer MP, Bellomo R, Cousins CE, Anninck CE, Sundararajan K, Reddi BAJ (2014). Dysglycaemia in the critically ill and the interaction of the chronic and acute glycaemia with mortality. Intensive Care Med..

[42] Macrea D, Grieve R, Allen E, Sadique Z, Morris K, Pappachan J, et al. A randomized trial of hyperglycemic control in pediatric intensive care. N Engl J Med. 2014;370:107–18. A published erratum appears in N Engl J Med. 2014;370:1469. 10.1056/NEJMoa1302564 24401049

